# USP30‐Mediated Deubiquitination of PEX5 Suppresses Pexophagy to Drive Tubular Injury in Diabetic Kidney Disease

**DOI:** 10.1002/advs.77161

**Published:** 2026-08-21

**Authors:** Jia Li, Chaoyang Hua, Guangpu Li, Furong Zhai, Jingjing Ren, Shaokang Pan, Qi Feng, Jianguo Wen, Dongwei Liu, Zhangsuo Liu, Jiayu Duan

**Affiliations:** ^1^ Research Institute of Nephrology Zhengzhou University The First Affiliated Hospital of Zhengzhou University Zhengzhou People's Republic of China; ^2^ Traditional Chinese Medicine Integrated Department of Nephrology The First Affiliated Hospital of Zhengzhou University Zhengzhou People's Republic of China; ^3^ Henan Province Research Center for Kidney Disease Zhengzhou People's Republic of China; ^4^ Key Laboratory of Precision Diagnosis and Treatment for Chronic Kidney Disease in Henan Province Zhengzhou People's Republic of China; ^5^ Department of Urology The First Affiliated Hospital of Zhengzhou University Zhengzhou People's Republic of China; ^6^ Department of Urology, Henan Children's Hospital Children's Hospital Affiliated to Zhengzhou University Zhengzhou People's Republic of China; ^7^ National Clinical Research Center for Kidney Diseases Jinling Hospital Nanjing Medical University Nanjing People's Republic of China

**Keywords:** diabetic kidney disease, peroxisome, pexophagy, ubiquitin‐specific peptidase 30

## Abstract

Diabetic kidney disease (DKD) is characterized by progressive tubular injury, yet the mechanisms linking metabolic stress to organelle dysfunction remain unclear. Here, utilizing human renal biopsies, db/db and high‐fat diet with streptozotocin‐induced diabetic mouse models, and cultured renal tubular epithelial cells (RTECs) exposed to 30 mM glucose, we demonstrate that impaired pexophagy drives peroxisomal dysfunction and tubular damage in DKD. Diabetic conditions induced marked accumulation of the peroxisomal membrane protein PMP70 (encoded by ABCD3/Abcd3) in RTECs, reflecting impaired peroxisomal turnover. We identified ubiquitin‐specific peptidase 30 (USP30) as a contributor of this process. Expression profiling and localization analyses revealed that USP30 expression was significantly elevated in vivo and in vitro under high‐glucose conditions and predominantly localized to RTECs. Global genetic depletion of USP30 restored peroxisomal function and metabolic homeostasis, enhanced pexophagy, and attenuated tubular injury. Mechanistically, USP30 antagonized the ubiquitination of the peroxisomal import receptor PEX5, thereby suppressing pexophagy. Furthermore, deletion of the essential autophagy factor ATG5 abolished the protective effects of USP30 deficiency. These findings reveal a critical role of USP30 mediated regulation of pexophagy in DKD and suggest that USP30 may serve as an experimental intervention target to alleviate tubular injury in DKD.

## Graphical abstract

USP30 accumulation in diabetic renal tubular epithelial cells deubiquitinates PEX5, suppresses pexophagy, and drives dysfunctional peroxisome accumulation and tubular injury. USP30 depletion restores PEX5 ubiquitination and pexophagy, improves peroxisomal function, and alleviates tubular damage, highlighting USP30 as a therapeutic target in diabetic kidney disease.

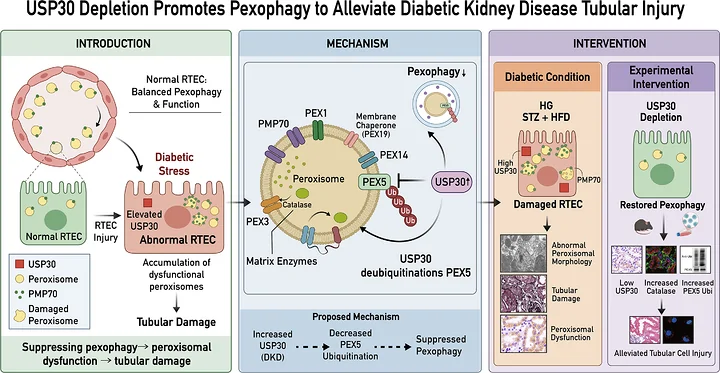

## 1 Introduction

Diabetic kidney disease (DKD) remains the leading cause of end‐stage renal disease worldwide and poses a substantial clinical challenge [1].Increasing evidence indicates that, beyond glomerular injury, renal tubular epithelial cells (RTEC) injury plays a critical role in DKD progression and closely correlates with declining renal function. Notably, tubular injury is increasingly recognized as an early event in DKD and may contribute to disease progression from the initial stages [2]. However, the mechanisms underlying tubular injury remain incompletely understood.

Given the high dependence of tubular cells on oxidative metabolism, organelles involved in redox homeostasis have gained increasing attention in DKD. Peroxisomes are essential metabolic organelles that participate in fatty acid oxidation and reactive oxygen species (ROS) metabolism [3]. Recent studies reported that impaired peroxisomal function and disrupted peroxisome related metabolism contribute to tubular injury and fibrotic progression in diabetic nephropathy [4, 5]. These observations imply that peroxisome quality control may represent an underexplored protective pathway in DKD. However, the mechanisms by which peroxisomal dysfunction drives tubular injury in DKD remain insufficiently understood.

In renal tubular cells, peroxisomal homeostasis is maintained by a delicate balance between the formation of new peroxisomes and selective autophagic degradation of dysfunctional peroxisomes (pexophagy) [6]. Pexophagy can be induced by multiple cellular stresses, including oxidative stress and nutrient deprivation [7]. During ubiquitin‐dependent pexophagy, the peroxisomal E3 ubiquitin ligase PEX2 ubiquitinates peroxisomal proteins, such as the peroxisomal import receptor PEX5 [8] and the membrane protein PMP70 (encoded by ABCD3/Abcd3) [9]. These ubiquitin signals are recognized by the selective autophagy receptors NBR1 and p62, which subsequently deliver peroxisomes to the autophagy‐lysosome system [10]. Under oxidative stress, ROS‐activated ATM phosphorylates PEX5 at Ser141, which promotes its monoubiquitination at Lys209 and facilitates receptor‐mediated pexophagy [11]. However, the role and regulation of this pathway in DKD remain to be clarified.

Deubiquitinating enzymes (DUBs) have been identified as key modulators of organelle specific autophagy by removing ubiquitin signals [12]. Accumulating evidence has established that ubiquitin‐specific protease 30 (USP30), which localizes to the outer mitochondrial membrane, acts a critical negative regulator of PINK1/Parkin‐mediated mitophagy to maintain mitochondrial fidelity [13, 14]. Beyond its role in mitochondrial quality control, emerging studies have recently hinted at its dual‐targeting capacity to peroxisomes, where it participates in basal organelle turnover by restraining pexophagy [15, 16]. However, whether USP30 acts as a molecular bridge mediating peroxisomal homeostatic networks under metabolic stress remains unexplored, particularly in the pathogenesis of DKD. The peroxisomal import receptor PEX5 has been recognized as a key regulator of this process, as its ubiquitination facilitates peroxisomal turnover [8]. Therefore, determining whether USP30 mediated deubiquitination of PEX5 impairs pexophagy and thereby exacerbates tubular injury is of particular importance.

In this study, we investigated whether dysregulated pexophagy contributes to peroxisomal dysfunction and tubular injury in DKD and whether USP30 is involved in this process. We demonstrated that PMP70 was increased in RTECs under DKD conditions. Depletion of USP30 attenuated tubular injury by restoring peroxisomal function and metabolism homeostasis. Furthermore, we evaluated the association between USP30 and PEX5 ubiquitination and investigated whether the effects of USP30 depletion involved an ATG5‐dependent autophagic pathway. Notably, depletion of the autophagy factor ATG5 abolished the protective effects of USP30 knockdown. Collectively, our findings suggest that pexophagy is essential for maintaining peroxisomal homeostasis and increased USP30 expression under diabetic conditions may impair ubiquitin‐dependent peroxisomal turnover and thereby contribute to renal tubular injury.

## 2 Result

### 2.1 Peroxisome Accumulation Is Increased in RTECs of Patients With DKD and db/db Mice

**FIGURE 1:**
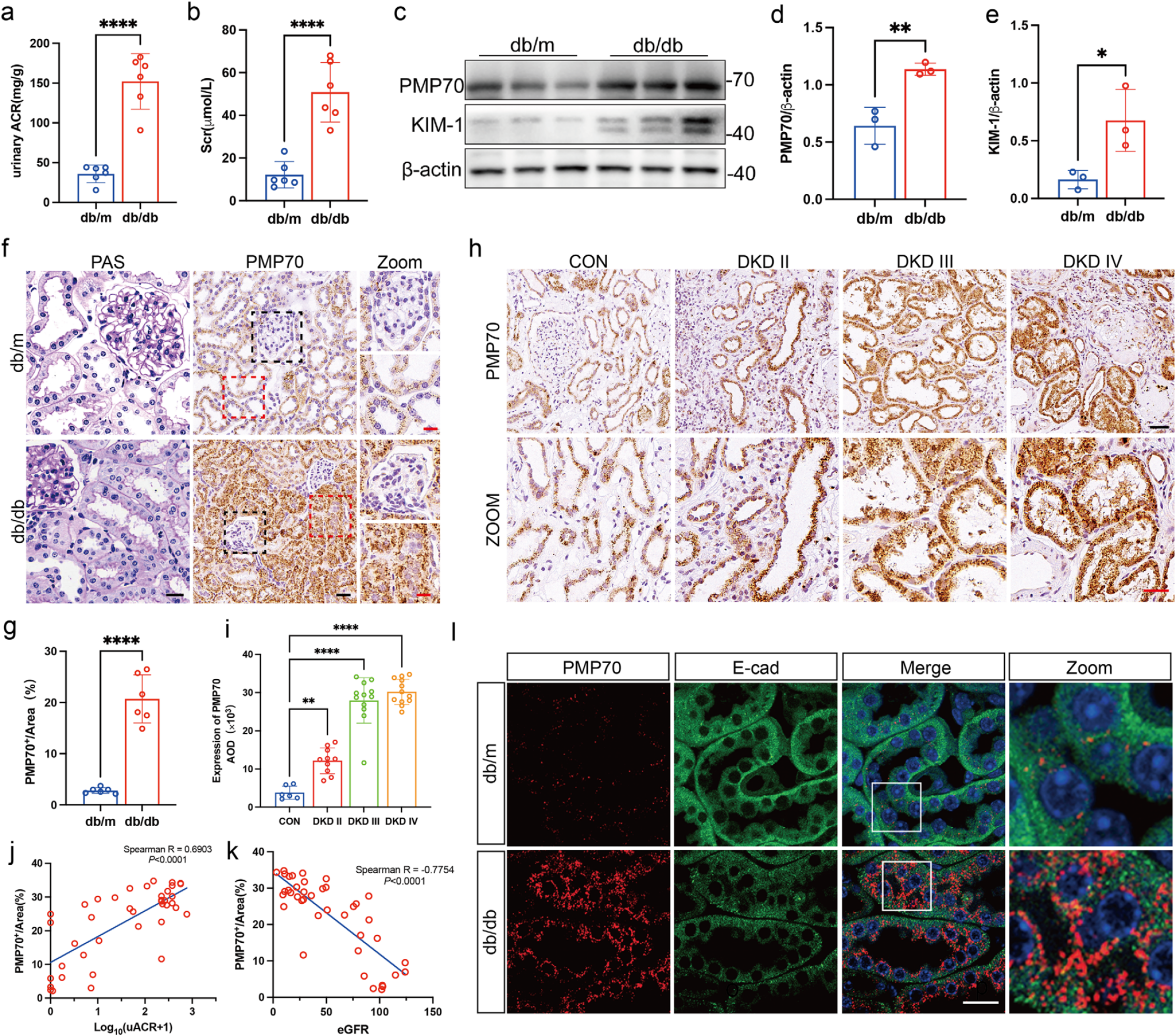
Peroxisome accumulation is increased in RTECs of patients with DKD and db/db mice. (a,b) Analysis of the urinary albumin/creatinine ratio (uACR) and serum creatinine (Scr) in db/db and db/m mice. n = 6. (c) Protein levels of the peroxisomal marker PMP70 and KIM‐1 were detected by western blot in db/db and db/m mice. (d,e) Quantification of PMP70 and KIM‐1 levels in db/db and db/m mice. n = 3. (f) Representative images of PAS staining and immunohistochemical (IHC) staining of PMP70 in kidney tissues from db/db and db/m mice. Black scale bar, 50 µm; red scale bar, 20 µm. (g) Quantification of PMP70 expression in IHC images from db/db and db/m mice. n = 6. (h,i) Representative IHC staining and quantification of PMP70 in renal biopsies from patients with DKD. Black scale bar, 50 µm; red scale bar,20 µm. (j,k) Correlation analysis between PMP70 staining intensity and uACR and eGFR from patients with DKD.uACR were transformed into Log_10_(uACR+1). n = 40. (l) Representative immunofluorescent staining for PMP70 (red) and E‐cadherin (green) in the kidney tissue from db/db and db/m mice (DAPI, blue). Scale bar, 10 µm. Data are presented as means ± SD. Statistical significance was determined by a two‐tailed unpaired Student's *t*‐test for two‐group comparisons, or by one‐way analysis of variance (ANOVA) followed by Tukey's post‐hoc test for multiple‐group comparisons. *p < 0.05, **p < 0.01, ***p < 0.001, ****p < 0.0001.

As shown in the schematic diagram of the sampling collection procedures of the mouse model (Figure S1a), we first evaluated the urinary albumin/creatinine ratio (uACR) and serum creatinine (Scr), of which db/db mice is significantly higher than that in db/m mice (Figure 1a,b, Figure S1b). Western blot analysis revealed a significant increase in PMP70 protein levels in kidneys from db/db mice, accompanied by elevated expression of kidney injury molecule‐1 (KIM‐1; HAVCR1/Havcr1), which is the marker of tubular injury (Figure 1c–e). Histological examination revealed that PMP70 protein was markedly increased in RTECs of db/db mice, whereas low expression was detected in glomeruli, indicating that peroxisomes are predominantly localized within the tubular compartment of kidney (Figure 1f,g, Figure S1c). To confirm these findings in human samples, we examined PMP70 expression in renal biopsy specimens from patients with DKD. Immunohistochemical (IHC) and histological staining demonstrated a significantly increased of PMP70 expression in DKD kidneys compared with controls (Figure 1h, Figure S1d). Notably, the expression of PMP70 protein levels was slightly upregulated in the DKD II stage (n = 10) but exhibited a more pronounced increase in both the DKD III (n = 12) and IV stages (n = 12) (Figure 1i). Consistently, the PMP70 expression level was positively correlated with the urinary albumin‐to‐creatinine ratio (uACR) and a negative correlation with eGFR (Figure 1j,k). Immunofluorescence staining further confirmed the tubular localization of PMP70 and demonstrated a significant increase in PMP70 fluorescence intensity in db/db mice, indicating enhanced peroxisome abundance in RTECs (Figure 1l, Figure S1e–g). In addition, PMP70 mRNA expression were examined. RNA sequencing and quantitative real‐time PCR (qRT‐PCR) revealed no significant differences between groups (Figure S1h,i). Collectively, these findings indicate that peroxisomes accumulate in RTECs of DKD patients and experimental models independently of transcriptional upregulation of PMP70, implicating dysregulated peroxisome turnover as a potential mechanism in DKD pathogenesis.

### 2.2 Accumulated Peroxisomes in RTECs Exhibit Functional Impairment and Defective Pexophagy

**FIGURE 2:**
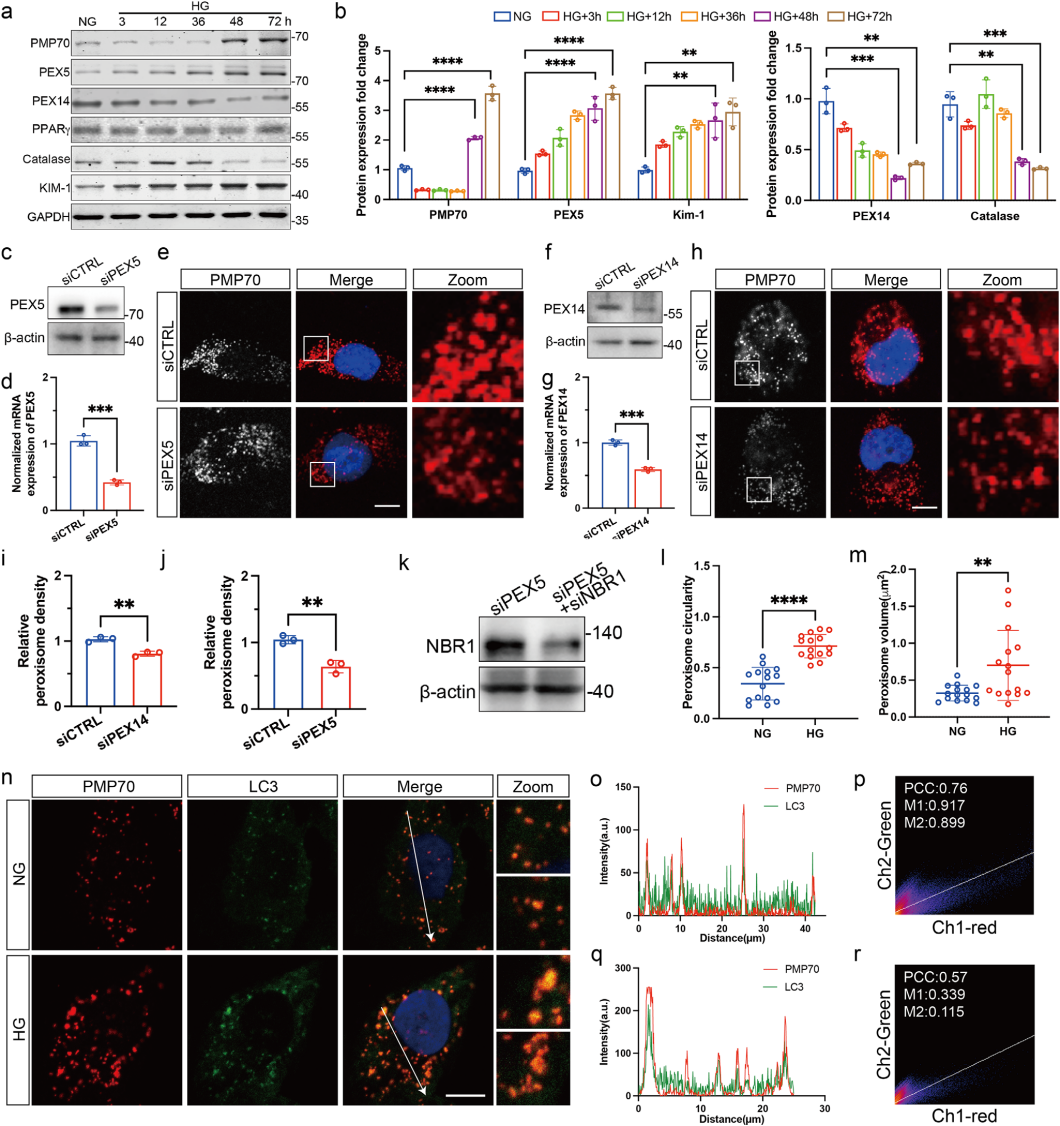
Accumulated peroxisomes in RTECs exhibit functional impairment and defective pexophagy. (a,b) Western blot analysis and quantification of key protein levels in HK‐2 cells treated with HG for different durations. n = 3. (c,d) Western blot and qRT‐PCR analyses of PEX5 expression in HK‐2 cells. n = 3. (e,j) Representative immunofluorescence staining for PMP70 (red) and quantification of peroxisome density in the siPEX5 and siCTRL groups. Scale bar, 10 µm. n = 3. (f,g) Western blot and qRT‐PCR analyses of PEX14 expression in HK‐2 cells. (h,i) Representative immunofluorescence staining for PMP70 (red) and quantification of peroxisome density in the siPEX14 and siCTRL groups. Scale bar, 10 µm. (k) Western blot analysis of NBR1 protein levels in HK‐2 cells. (l,m) Quantification of mean peroxisome circularity and volume in the images (n). Fifteen images from different microscopic fields were analyzed in each group. (n) Representative immunofluorescence staining for PMP70 (red) and LC3 (green) in kidney tissues from db/db and db/m mice (DAPI, blue). Scale bar, 10 µm. (o–r) Intensity of PMP70 and LC3 colocalization within the direction indicated by white arrows in (n) was measured, and Pearson's overlap coefficients were quantified in the groups shown in (n). Data are shown as means ± SD and were analyzed by one‐way ANOVA. **p < 0.01, ***p < 0.001, ****p < 0.0001.

To determine whether the accumulated peroxisomes observed in RTECs exhibit functional abnormalities and was associated with pexophagy, we examined key components involved in peroxisomal matrix protein import and peroxisome homeostasis, including the cargo receptor PEX5 and the membrane docking protein PEX14. HK‐2 cells were exposed to high glucose (HG, 30 mM), and the expression of peroxisome‐associated functional markers were analyzed at 3, 12, 36, 48, and 72 h. The significant changes were observed at 48 h, with marked peroxisome accumulation, increased PEX5 protein levels, and reduced PEX14 expression, accompanied by a significant decrease in the peroxisomal antioxidant enzyme catalase, indicating impaired peroxisomal function (Figure 2a,b). In contrast, expression of peroxisome proliferator–activated receptor γ (PPARγ), a key regulator of peroxisome biogenesis, remained unchanged, indicating that peroxisome accumulation was not attributable to enhanced biogenesis (Figure S2a).

To determine whether alterations in peroxisomal import machinery contribute to impaired peroxisome clearance, PEX5 and PEX14 were silenced by siRNA, with PEX3 and PEX19, two essential peroxisome biogenesis factors, knockdown serving as controls for biogenesis related effects. Peroxisome abundance was quantified using Z‐stack confocal imaging, and peroxisome density was calculated as the number of peroxisomes normalized to cell volume. Knockdown of PEX5 or PEX14 significantly reduced peroxisome density compared with control cells (Figure 2c–j), whereas silencing of PEX3 or PEX19 did not result in a significant reduction in peroxisome number (Figure S2b–i). Furthermore, knockdown of PEX5 or PEX14 significantly reduced catalase activity, further indicating impaired peroxisomal function (Figure S2j,k). Given that peroxisomes are primarily degraded through selective autophagy, we further examined the role of pexophagy by silencing the selective autophagy receptor NBR1, which mediates the recognition and sequestration of ubiquitinated peroxisomes into forming autophagosomes. Notably, knockdown of NBR1 prevented the loss of peroxisomes induced by PEX5 depletion, indicating that peroxisome reduction under these conditions was dependent on pexophagy (Figure 2k; Figure S2l,m).

Consistently, immunofluorescence analysis of PMP70 and LC3 further revealed that HG treatment was associated with increased peroxisome volume and circularity, indicative of enlarged and abnormal peroxisomal morphology (Figure 2l,m). Moreover, PMP70/LC3 colocalization was significantly reduced, suggesting impaired recruitment of peroxisomes to autophagosomes (Figure 2n–r). To further assess pexophagy‐associated lysosomal delivery, we performed additional immunofluorescence colocalization analysis of PMP70 with the lysosomal marker LAMP1. Compared with NG conditions, HG treatment reduced PMP70/LAMP1 colocalization, indicating decreased delivery of peroxisomes to lysosomes (Figure S2n). In line with these observations, analysis of autophagic related proteins further supported defective pexophagy under HG conditions (Figure S2o,p). Together, these results indicate that impaired pexophagy contributes to the accumulation of dysfunctional peroxisomes in HK‐2 cells.

### 2.3 Upregulation of USP30 Is Associated With Reduced Ubiquitination‐Dependent PEX5 Degradation

**FIGURE 3:**
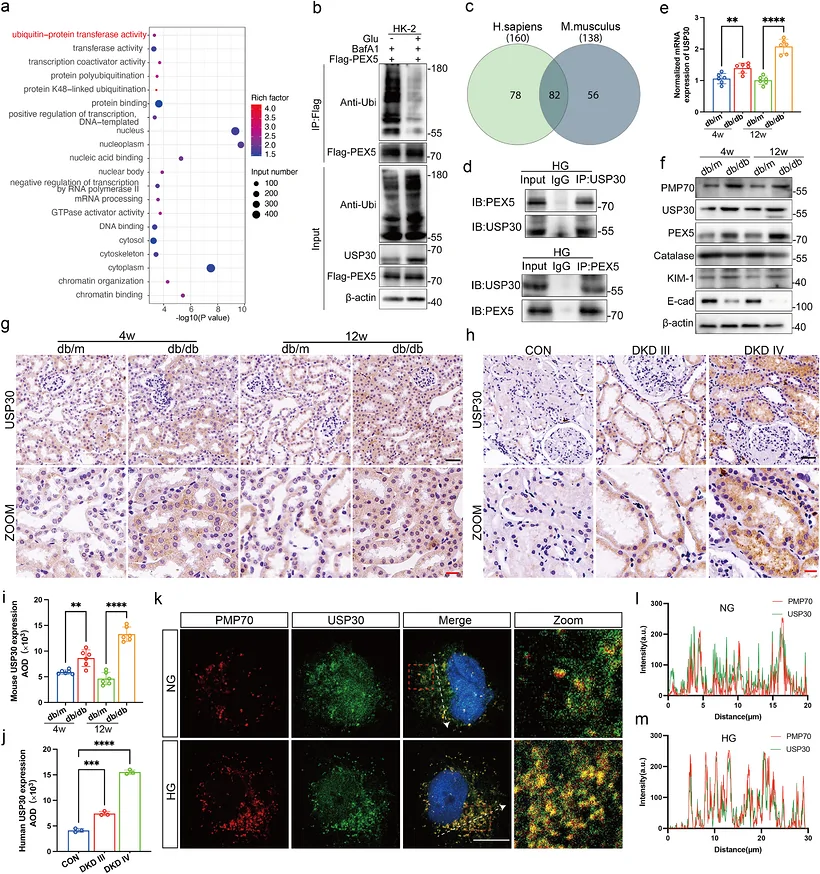
Upregulation of USP30 is associated with reduced ubiquitination‐dependent PEX5 degradation. (a) GO analysis of the biological processes involved in the predicted transcription pathways. (b) Co‐IP analysis of ubiquitination of PEX5 in HK‐2 cells transfected with plasmids as indicated. (c) A Venn diagram showing potential ligase that could bind to the PEX5 in both human and mouse datasets. (d) Co‐IP analysis of the interaction between endogenous PEX5 and USP30. (e) qRT‐PCR analysis of USP30 expression in db/db and db/m mice. n = 6. (f) Western blot analysis of PMP70, USP30, PEX5, Catalase, KIM‐1 and E‐cadherin protein levels in the kidney tissues from db/db and db/m mice. (g) Representative images of IHC staining of USP30 in kidney tissues from db/db and db/m mice and (h) renal biopsies from patients with DKD. Black scale bar, 50 µm; red scale bar, 20 µm. (i) Quantification of USP30 expression in mouse IHC images. n = 6. (j) Quantification of USP30 expression in human IHC images. n = 3. (k) Representative immunofluorescence staining for PMP70 (red) and USP30 (green) in HK‐2 cells (DAPI, blue). Scale bar, 10 µm. (l,m) Intensity of PMP70 and USP30 colocalization within the direction indicated by white arrows in (k) was measured. Data are presented as means ± SD. Statistical analyses for (e,i) were performed using two‐way ANOVA followed by Bonferroni's post‐hoc test for pairwise comparisons at each time point, for (j) was performed using one‐way ANOVA followed by Tukey's post‐hoc test. **p < 0.01, ***p < 0.001, ****p < 0.0001.

To further elucidate the molecular mechanisms underlying impaired pexophagy during DKD progression, transcriptomic profiling was performed on kidney tissues from db/db and control mice. Gene Ontology (GO) and Kyoto Encyclopedia of Genes and Genomes (KEGG) enrichment analyses revealed significant enrichment of ubiquitin‐protein related pathways (Figure 3a; Figure S3a). Given that accumulation of ubiquitinated PEX5 at the peroxisomal membrane promotes pexophagy, we next examined the ubiquitination status of PEX5. In contrast with increased PEX5 protein abundance, ubiquitination of PEX5 was markedly reduced in HG induced HK‐2 cells (Figure 3b).

To further identify potential ubiquitin‐system regulators of PEX5, we used UbiBrowser, which provides both known and predicted E3 ubiquitin ligase‐substrate interactions and deubiquitinase‐substrate interactions [17], to predict DUBs that may be involved in PEX5 regulation in both human and mouse datasets (Figure 3c). Among the predicted candidates, deubiquitinating enzymes ubiquitin‐specific peptidase 30 (USP30) was identified as a candidate DUB potentially associated with PEX5 regulation. Previous studies also demonstrated that USP30 localizes to peroxisomes and restrains basal pexophagy [16], and that USP30 depletion increases the ubiquitination of the peroxisomal membrane proteins PEX5 and PMP70 [15]. Reciprocal co‐immunoprecipitation (Co‐IP) assays further confirmed the interaction between USP30 and PEX5 (Figure 3d).

For further verification, 10‐week‐old db/db mice were analyzed after 4 and 12 weeks (Figure S3b). Compared with control mice, db/db mice exhibited significant increases in body weight, uACR, blood glucose levels, and kidney weight (Figure S3c–f). qRT‐PCR demonstrated that renal USP30 mRNA expression was significantly increased in db/db mice and progressively upregulated with severity of injury (Figure 3e). Consistently, Western blot analysis revealed increased protein levels of USP30, PEX5, and PMP70, accompanied by reduced catalase expression in both 4 and 10 weeks (Figure 3f; Figure S3g–l). IHC analysis further confirmed the levels of USP30 expression were elevated in tubular cells from db/db mice (Figure 3g,i) and DKD patients (Figure 3h,j), with USP30 levels increasing with disease progression. Moreover, we measured cytoplasmic colocalization of USP30 with the peroxisomal marker PMP70 in RTECs. Immunofluorescence analysis revealed that USP30 colocalized with PMP70, and this colocalization was increased under HG conditions (Figure 3k–m). Collectively, these findings suggest that USP30 upregulation is associated with reduced PEX5 ubiquitination and impaired pexophagy, thereby contributing to peroxisome accumulation during DKD progression.

### 2.4 Overexpression of USP30 Exacerbates Peroxisome Accumulation and Impairs Pexophagy in db/m Mice

**FIGURE 4:**
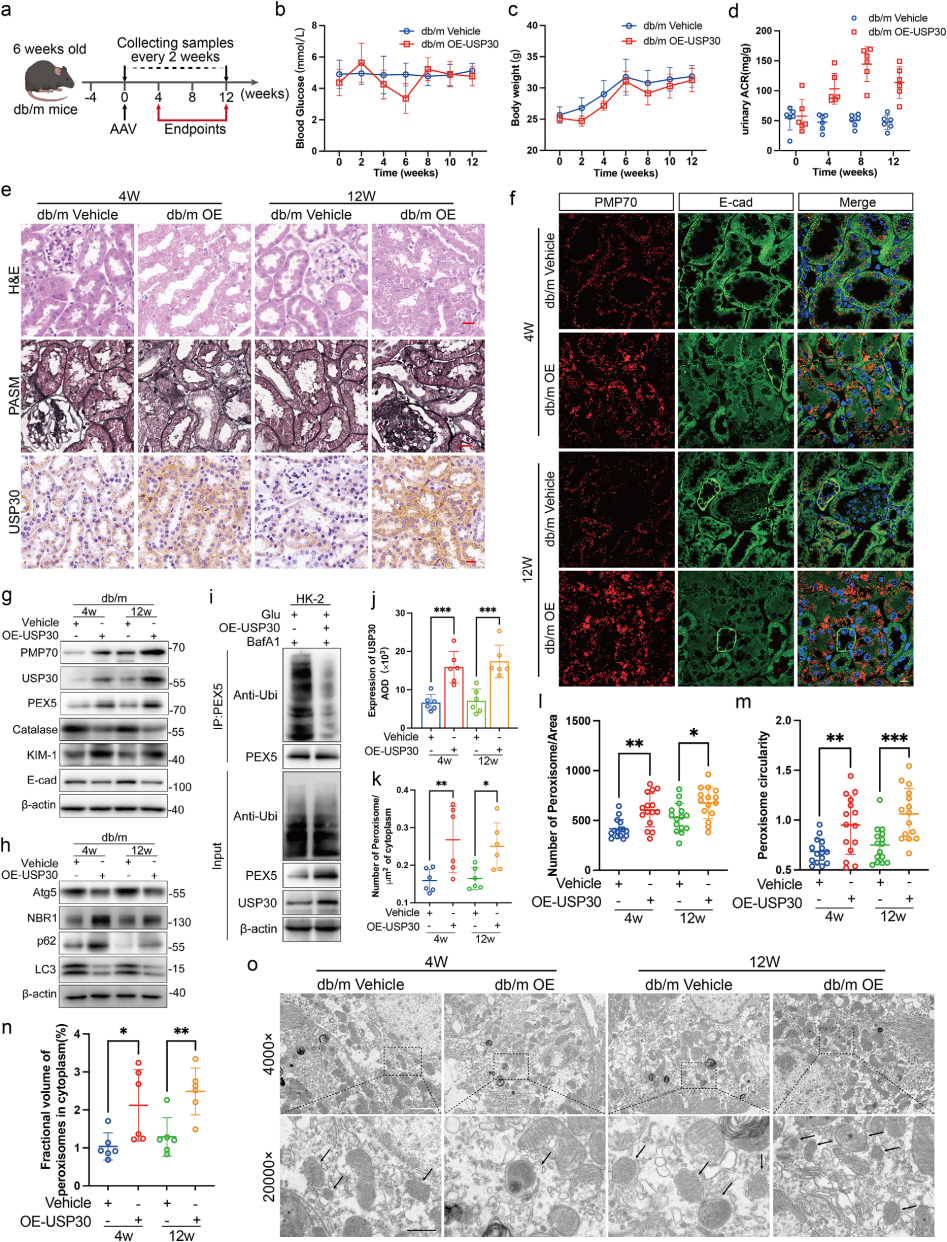
Overexpression of USP30 exacerbates peroxisome accumulation and impairs pexophagy in db/m mice. (a) Schematic diagram of the treatment and sampling collection procedures of the mouse model. (b–d) Blood glucose, body weight, uACR in vehicle and overexpression of USP30 db/m mice. n = 6 mice/group. (e) Representative images of H&E staining, Periodic Acid‐Methenamine Silver (PASM) staining, and IHC staining of USP30 in kidney tissues from db/m mice. Red scale bar, 20 µm. (f) Representative immunofluorescent staining for PMP70 (red) and E‐cadherin (green) in the kidney tissues from db/m mice (DAPI, blue). Scale bar, 10 µm. (g) Western blot analysis of PMP70, USP30, PEX5, Catalase, KIM‐1 and E‐cadherin protein levels in the kidney tissues from db/m mice. (h) Western blot analysis of Atg5, NBR1, p62 and LC3 in the kidney tissues from db/m mice. (i) Co‐IP analysis of PEX5 ubiquitination in HK‐2 cells. (j) Quantification of USP30 expression in the IHC images shown in (e). (k,n) Quantification of mean peroxisome number and fractional volume in the images (o). Six images from different microscopic fields were analyzed in each group. (l,m) Quantification of mean peroxisome circularity and number in the images (f). Fifteen images from different microscopic fields were analyzed in each group. (o) Representative TEM images showing peroxisomal morphology and number in kidney tissues from db/m mice. Scale bars, 2 µm (4000×) and 500 nm (12 000×). Data are presented as means ± SD. Statistical analyses for (b–d) were performed using two‐way repeated measures ANOVA followed by Bonferroni's post‐hoc test. For (j–n), statistical analyses were performed using two‐way ANOVA followed by Bonferroni's post‐hoc test for pairwise comparisons within each row. *p < 0.05, **p < 0.01, ***p < 0.001.

To investigate whether USP30 overexpression promotes peroxisome accumulation and impairs pexophagy in vivo, an adeno‐associated virus serotype 9 vector driving USP30 expression under the proximal tubule‐specific Ksp‐cadherin promoter (AAV9‐USP30‐OE) was generated (Figure S4a) and administered via tail‐vein injection to 10‐week‐old db/m mice (Figure 4a). At 4 and 12 weeks after injection, the blood glucose, body weight, or kidney weight (Figure 4b,c; Figure S4b) were not significantly difference between the USP30‐OE and vector mice. However, overexpression of USP30 significantly increased the uACR (Figure 4d) and aggravated renal injury (Figure 4e; Figure S4d). IHC and qRT‐PCR confirmed the efficient transduction with the upregulation of USP30 in RTECs (Figure 4e,j; Figure S4c). Immunofluorescence analysis further showed that USP30 overexpression was associated with increased peroxisome abundance, together with increased peroxisome circularity, which indicated the peroxisome were accumulated and have morphological abnormalities (Figure 4f,l,m).

Western blotting demonstrated the stable induction of USP30 at 4 to 12 weeks after injection. The PMP70, USP30 and PEX5 levels were notably increased, reduced E‐cadherin expression, and elevated KIM‐1, indicating exacerbated tubular injury (Figure 4g; Figure S4e–j). Moreover, in HG‐induced HK‐2 cells, we observed a reduction in the ubiquitination level of PEX5 (Figure 4i), suggesting that USP30 may stabilize PEX5 through its deubiquitinating regulation. Meanwhile, the expression of catalase was decreased (Figure 4g), and immunofluorescence staining analysis revealed a reduced catalase fluorescence intensity (Figure S4o,p), indicating impaired peroxisomal function. Following USP30 overexpression, the levels of the autophagy‐related proteins ATG5 and LC3 were reduced, whereas p62 and NBR1 were increased, indicating suppression of autophagic flux and thereby promoting further accumulation of aberrant peroxisomes (Figure 4h; Figure S4k–n). Consistent with these results, Periodic Acid‐Methenamine Silver (PASM) and transmission electron microscopy (TEM) revealed that the RTECs in USP30‐OE mice exhibited cellular hypertrophy and swelling compared to control mice. In addition, the number of peroxisomes within RTECs was increased, accompanied by evident morphological abnormalities (Figure 4k,n,o). Collectively, these findings demonstrate that overexpression of USP30 exacerbates peroxisome accumulation and impairs pexophagy in db/m mice, accompanied by peroxisomal dysfunction and aggravated tubular injury.

### 2.5 USP30 Knockout Alleviates Tubular Injury in DKD Mice by Restores Peroxisomal Dysfunction and Facilitates Pexophagy

**FIGURE 5:**
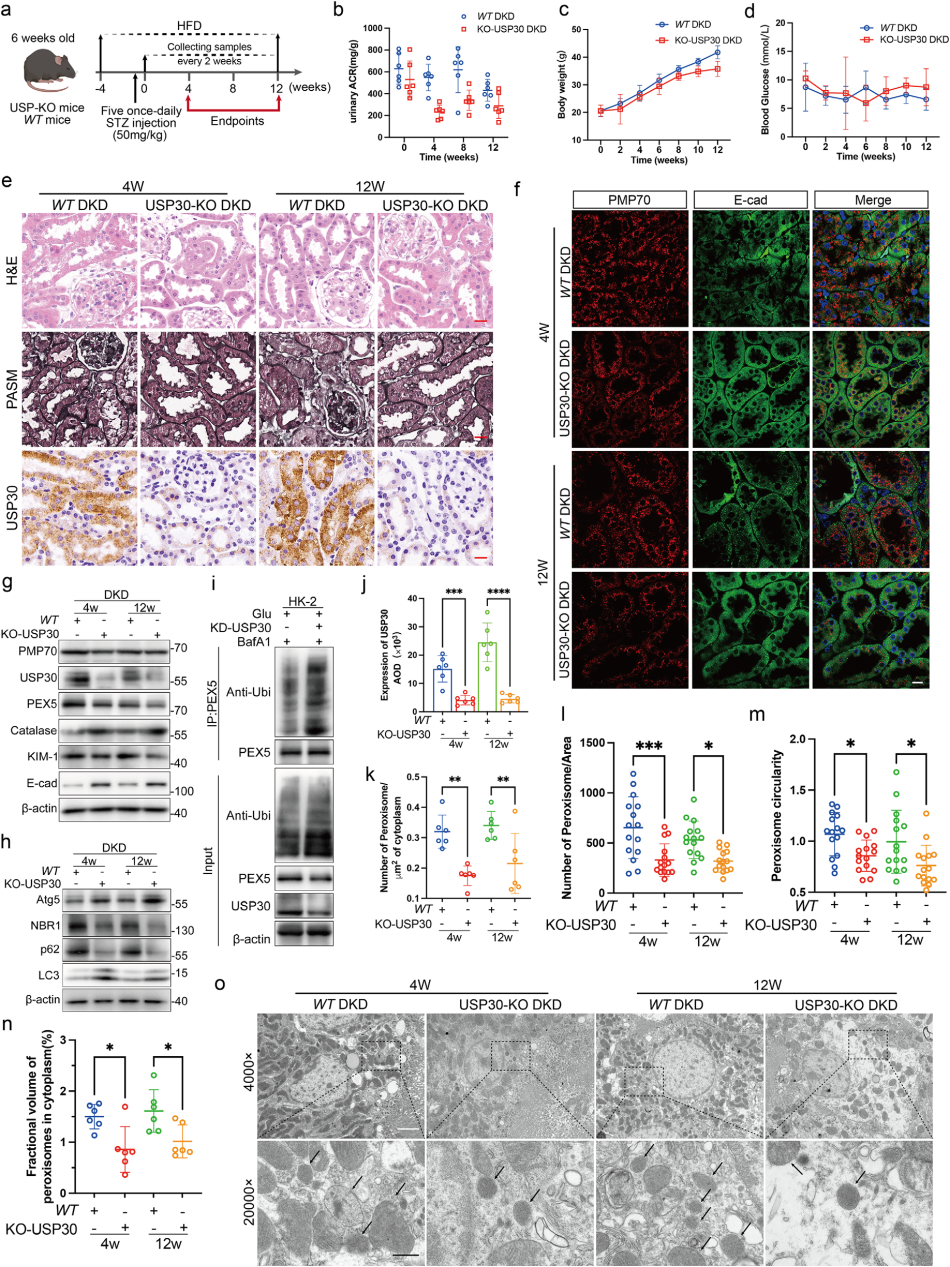
USP30 knockout alleviates tubular injury in DKD mice by restoring peroxisomal function and facilitating pexophagy. (a) Schematic diagram of the treatment and sampling collection procedures of the mouse model. (b–d) uACR, blood glucose, body weight, in WT and USP30 knockout mice. n = 6 mice/group. (e) Representative images of H&E staining, PASM staining, and IHC staining of USP30 in kidney tissues from DKD mice. Red scale bar, 20 µm. (f) Representative immunofluorescent staining for PMP70 (red) and E‐cadherin (green) in the kidney tissues from DKD mice (DAPI, blue). Scale bar, 10 µm. (g) Western blot analysis of PMP70, USP30, PEX5, catalase, KIM‐1 and E‐cadherin protein levels in the kidney tissues from DKD mice. (h) Western blot analysis of Atg5, NBR1, p62 and LC3 in the kidney tissues from DKD mice. (i) Co‐IP analysis of PEX5 ubiquitination in HK‐2 cells. (j) Quantification of USP30 expression in the IHC images shown in (e). (k,n) Quantification of mean peroxisome number and fractional volume in the images (o). Six images from different microscopic fields were analyzed in each group. (l,m) Quantification of mean peroxisome circularity and number in the images (f). Fifteen images from different microscopic fields were analyzed in each group. (o) Representative TEM images showing peroxisomal morphology and number in kidney tissues from db/db mice. Scale bars, 2 µm (4000×) and 500 nm (12 000×). Data are presented as means ± SD. Statistical analyses for (b—d) were performed using two‐way repeated measures ANOVA followed by Bonferroni's post‐hoc test. For (j—n), statistical analyses were performed using two‐way ANOVA followed by Bonferroni's post‐hoc test for pairwise comparisons within each row. *p < 0.05, **p < 0.01, ***p < 0.001, ****p < 0.0001.

To determine whether USP30 knockout is sufficient to ameliorate diabetes‐induced tubular injury, USP30‐KO mice were generated using the CRISPR/Cas9 technology and genotypes were confirmed by tail gene (Figure S5a). Then, both USP30‐KO mice and Wild‐Type (WT) littermate mice were treatment with streptozotocin (STZ) and feeding a high‐fat diet (HFD) to induce DKD (Figure 5a). Compared with WT DKD mice, USP30 inhibition significantly reduced the uACR (Figure 5b) and alleviated tubular injury (Figure 5e; Figure S5b). In contrast, USP30 depletion did not significantly affect blood glucose levels, body weight, or kidney weight (Figure 5c,d; Figure S5c). IHC staining and qRT‐PCR analysis confirmed the efficient downregulation of USP30 in RTECs of DKD mice (Figure 5e,j; Figure S5d). Immunofluorescence analysis further revealed that USP30 knockout was associated with restoration of E‐cadherin expression, accompanied by a reduction in peroxisome abundance and decreased peroxisomal circularity, indicating attenuation of abnormal peroxisome accumulation and improved morphological uniformity (Figure 5f,l,m).

Western blotting analysis demonstrated the levels of the peroxisomal membrane protein PMP70 and PEX5 were significantly decreased, whereas E‐cadherin expression was restored and KIM‐1 was reduced in USP30‐KO mice (Figure 5g; Figure S5e–j). In HG‐induced HK‐2 cells, USP30 knockdown led to a markedly increase in the ubiquitination level of PEX5 (Figure 5i), suggesting enhanced autophagic turnover of PEX5 following USP30 depletion. Meanwhile, catalase expression was markedly increased (Figure 5g), and immunofluorescence staining confirmed an elevation in catalase fluorescence intensity (Figure S5o,p), indicating restoration of peroxisomal function. In parallel, USP30 knockout promoted autophagic activity, as evidenced by increased expression of the autophagy‐related proteins ATG5 and LC3, along with decreased levels of p62 and NBR1, facilitating the autophagic clearance of aberrant peroxisomes (Figure 5h; Figure S5k–n). PASM and TEM revealed that RTECs in USP30‐KO mice exhibited attenuated cellular hypertrophy and structural injury compared with control mice. Moreover, the number of peroxisomes was reduced, accompanied by decreased morphological heterogeneity (Figure 5k,n,o). Collectively, these results demonstrate that knockout of USP30 alleviates tubular injury in DKD mice by reducing the accumulation of dysfunctional peroxisomes and restoring pexophagy and peroxisomal function.

### 2.6 USP30 Knockdown Enhances Pexophagy and Ameliorates Tubular Injury In Vitro

**FIGURE 6:**
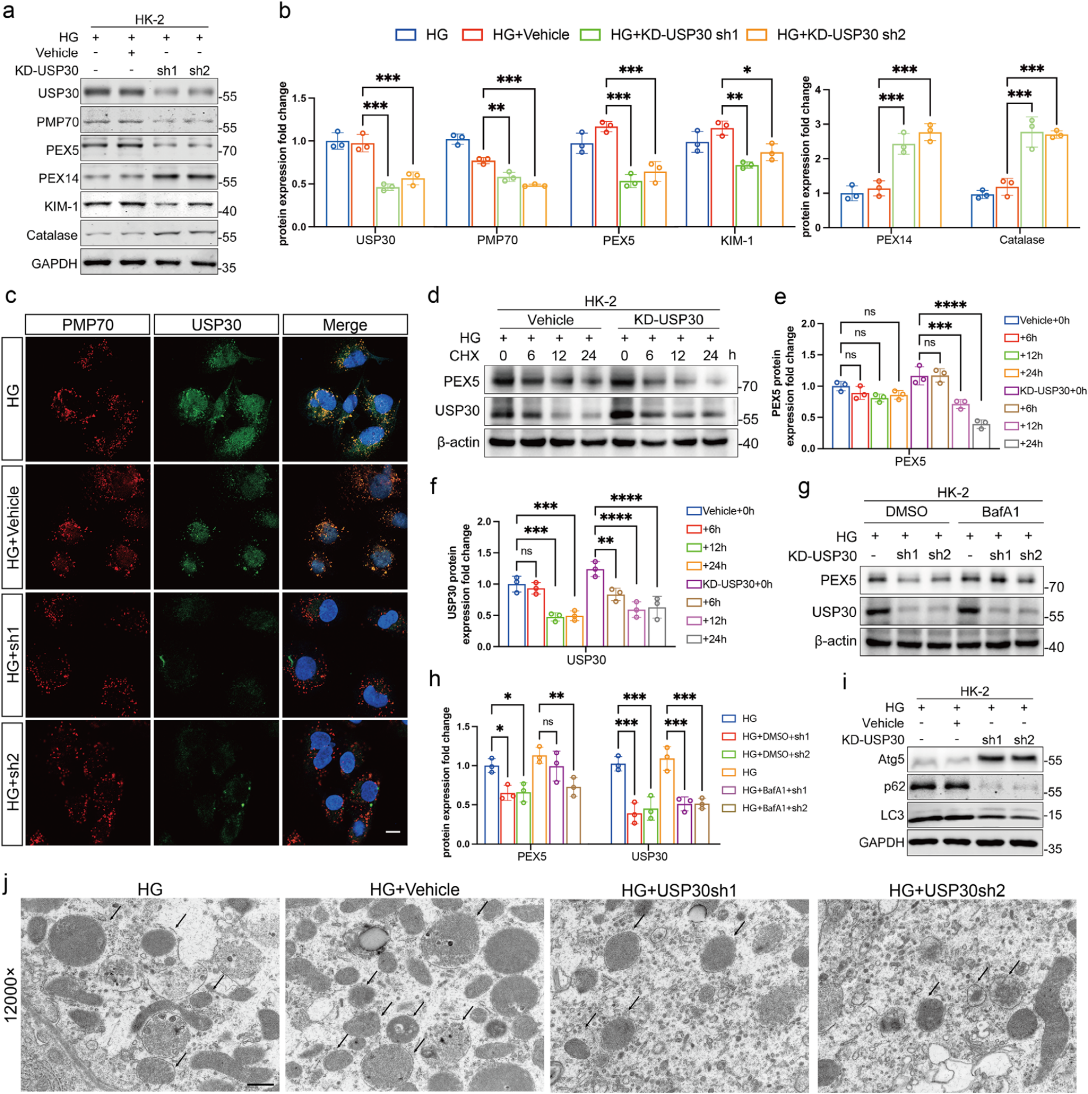
USP30 knockdown enhances pexophagy and ameliorates tubular injury in HG‐induced HK‐2 cells. (a,b) Western blot analysis and quantification of USP30, PMP70, PEX5, PEX14, KIM‐1, and catalase protein levels in HK‐2 cells under the indicated conditions. (c) Representative immunofluorescence staining for PMP70 (red) and USP30 (green) in HK‐2 cells under the indicated conditions. (DAPI, blue). Scale bar, 10 µm. (d–f) Western blot analysis and quantification of PEX5 and USP30 protein levels in HK‐2 cells under the indicated conditions. (g,h) Western blot analysis and quantification of PEX5 and USP30 protein levels in HK‐2 cells under the indicated conditions. *n* = 3. (i) Western blot analysis of Atg5, p62 and LC3 in HK‐2 cells under the indicated conditions. *n* = 3. (j) Representative TEM images showing peroxisomal morphology and number in HK‐2 cells under the indicated conditions. Scale bars, 500 nm (12 000×). Data are presented as means ± SD. For (b), the statistical significance was determined independently by one‐way ANOVA followed by Tukey's post‐hoc test for multiple pairwise comparisons. (e,f) were performed using two‐way ANOVA followed by Bonferroni's post‐hoc test for pairwise comparisons between the vehicle and KD‐USP30 groups at each designated time point (0, 6, 12, and 24 h). (g) were determined by two‐way ANOVA followed by Tukey's post‐hoc test for multiple pairwise comparisons among the control and shRNA groups within each treatment condition (DMSO or BafA1). *p < 0.05, **p < 0.01, ***p < 0.001, ****p < 0.0001. ns, not significant.

To further investigate whether USP30 plays a critical role in HG induced peroxisomal dysfunction, USP30 was knocked down in HG‐induced HK‐2 cells via shRNA lentiviral transduction. qRT‐PCR analysis showed that knockdown of USP30 did not significantly affect the mRNA levels of PMP70, indicating that USP30 may not directly impact peroxisome biogenesis, consistent with our previous observations (Figure S6a,b). Western blot analysis revealed that, compared with the HG and HG+Vehicle cells, USP30 depletion significantly reduced the accumulation of PEX5 and increased PEX14 expression, along with decreased expression of KIM‐1. Conversely, the antioxidant enzyme catalase was upregulated, supporting improved peroxisomal function following USP30 knockdown (Figure 6a,b). To further assess the specificity of USP30 knockdown, we re‐expressed wild‐type USP30 in shUSP30 HK‐2 cells. Re‐expression of USP30 partially reversed the effects of USP30 knockdown on peroxisomal markers and tubular injury proteins, supporting that these changes were associated with USP30 depletion (Figure S6c).

Immunofluorescence staining further confirmed that USP30 knockdown decreased PMP70 positive puncta and reduced peroxisomal abundance in HG‐induced cells (Figure 6c). Additionally, protein synthesis was blocked using cycloheximide (CHX), and pulse‐chase analysis revealed that USP30 knockdown significantly shortened the half‐life of PEX5 protein in HK‐2 cells (Figure 6d–f), thereby promoting PEX5 ubiquitination and facilitating its entry into the degradation pathway.

To further examine that PEX5 degradation occurs through the autophagy‐lysosome pathway, cells were treated with the lysosomal inhibitor bafilomycin A1 (BafA1) following shUSP30 transfection under HG stimulation. Notably, BafA1 treatment prevented the reduction of PEX5 levels, indicating that USP30 depletion facilitates lysosome dependent degradation of PEX5 and supports the involvement of pexophagy (Figure 6g,h). Next, pexophagy related proteins including p62, ATG5, and LC3 were examined. USP30 knockdown increased ATG5 expression and the LC3‐II/I ratio, while reducing p62 levels (Figure 6i, Figure S6d), indicating enhanced autophagic flux and activation of peroxisome turnover via pexophagy. Consistently, TEM revealed a reduced number of peroxisomes in HG+shUSP30 cells compared with HG‐induced cells (Figure 6j). Morphological analysis further showed improved peroxisomal integrity and more uniform organelle size, supporting the restoration of peroxisomal homeostasis (Figure S6e,f). These findings demonstrate that USP30 knockdown promotes pexophagy mediated clearance of dysfunctional peroxisomes and alleviates tubular cell injury.

### 2.7 USP30 Overexpression Impaired Pexophagy and Aggravated Tubular Injury In Vitro

**FIGURE 7:**
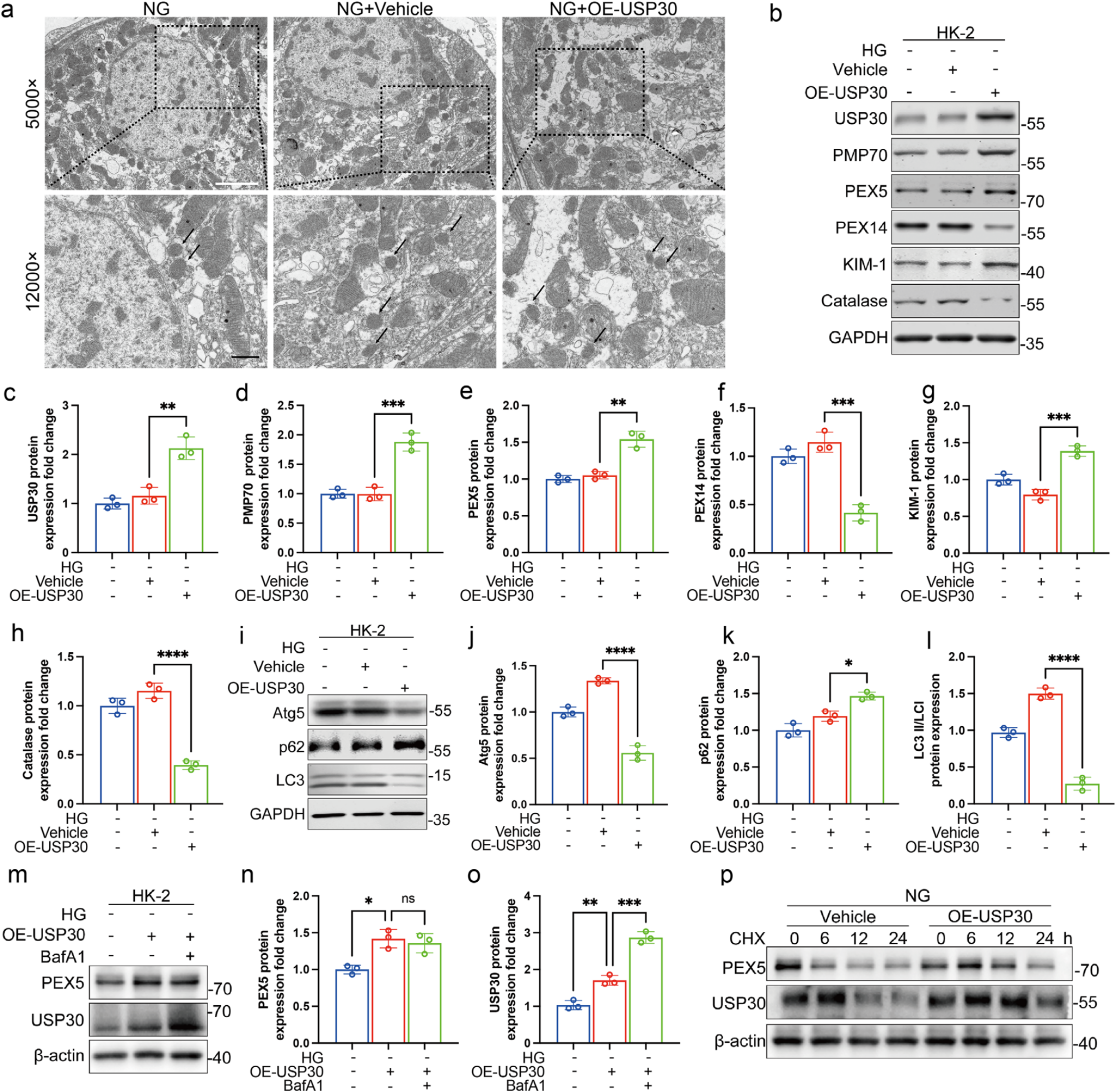
USP30 overexpression impaired pexophagy and aggravated tubular injury in HK‐2 cells. (a) Representative TEM images showing peroxisomal morphology and number in HK‐2 cells under the indicated conditions. Scale bars, 2 µm (5000×), 500 nm (12 000×). (b–h) Western blot analysis and quantification of USP30, PMP70, PEX5, PEX14, KIM‐1, and catalase protein levels in HK‐2 cells under the indicated conditions. (i–l) Western blot analysis and quantification of Atg5, p62 and LC3 protein levels in HK‐2 cells under the indicated conditions. (m–o) Western blot analysis and quantification of PEX5 and USP30 protein levels in HK‐2 cells treated with BafA1. (p) Western blot analysis of PEX5 and USP30 protein levels in HK‐2 cells treated with CHX for the indicated durations. Data are presented as means ± SD. Statistical analyses were performed by one‐way ANOVA followed by Tukey's post‐hoc test for multiple pairwise comparisons among the three experimental groups. *p < 0.05, **p < 0.01, ***p < 0.001, ****p < 0.0001, ns, not significant.

Based on the above findings that USP30 participates in PEX5‐mediated pexophagy, we next examined whether USP30 overexpression could induce tubular injury in NG‐treated HK‐2 cells. The overexpression efficiency was confirmed (Figure S7a). TEM showed that USP30 overexpression led to abnormal peroxisomal morphology and increased peroxisome abundance (Figure 7a, Figure S7b,c). Compared with NG and NG+vehicle cells, the protein levels of PMP70 and PEX5 were elevated, whereas PEX14 and catalase were reduced. In parallel, the tubular injury marker KIM‐1 was markedly upregulated, indicating aggravated cellular damage (Figure 7b–h). Moreover, USP30 overexpression suppressed pexophagy flux, as reflected by decreased ATG5 expression and LC3‐II/I ratio, whereas the p62 accumulation was increased (Figure 7i–l). Treatment with the lysosomal inhibitor BafA1 further increased PEX5 accumulation in USP30 overexpression cells (Figure 7m–o), indicating that PEX5 turnover was blocked at the lysosomal stage. In addition, CHX chase analysis, performed to block de novo protein synthesis, demonstrated that USP30 overexpression prolonged the stability of PEX5 protein (Figure 7p), supporting that PEX5 degradation was delayed upon USP30 mediated deubiquitination.

### 2.8 ATG5‐Dependent Pexophagy Is Required for the Protective Effects of USP30 Knockdown in HK‐2 Cells

**FIGURE 8:**
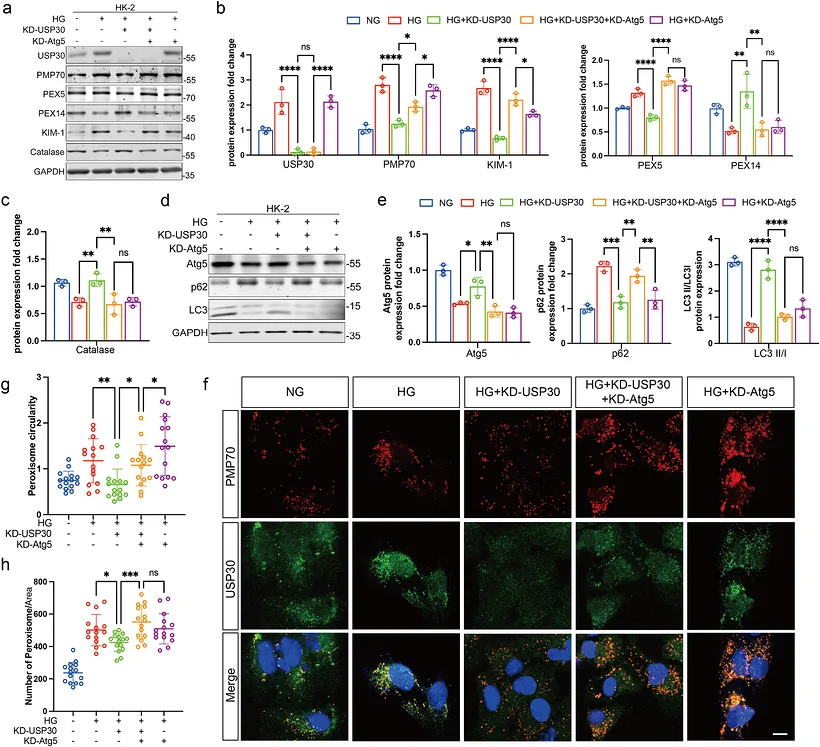
ATG5‐dependent pexophagy is required for the protective effects of USP30 knockdown in HK‐2 cells. (a–c) Western blot analysis and quantification of USP30, PMP70, PEX5, PEX14, KIM‐1, and catalase protein levels in HK‐2 cells under the indicated conditions. (d,e) Western blot analysis and quantification of Atg5, p62 and LC3 protein levels in HK‐2 cells under the indicated conditions. (f) Representative immunofluorescence staining for PMP70 (red) and USP30 (green) in HK‐2 cells under the indicated conditions. (DAPI, blue). Scale bar, 10 µm. (g,h) Quantification of mean peroxisome circularity and number in the images (f). Fifteen images from different microscopic fields were analyzed in each group. Data are shown as means ± SD and were analyzed by one‐way ANOVA followed by Tukey's post‐hoc test for multiple pairwise comparisons. *p < 0.05, **p < 0.01, ***p < 0.001, ****p < 0.0001, ns, not significant.

To determine whether the protective effects of USP30 knockdown under HG stress depend on ATG5 mediated pexophagy, ATG5 was further intervented in USP30 knockdown HK‐2 cells. Western blot analysis showed that USP30 knockdown reduced the accumulation of peroxisomal markers PMP70 and PEX5, restored PEX14 and catalase expression, and attenuated tubular injury, as evidenced by decreased KIM‐1 levels (Figure 8a–c). Notably, these beneficial effects were reversed when ATG5 was simultaneously knocked down, leading to re‐accumulation of peroxisomal proteins and re‐elevation of KIM‐1 expression. In parallel, ATG5 knockdown alone also resulted in the peroxisomal accumulation, further supporting impaired peroxisomal turnover (Figure 8a–c). Moreover, USP30 knockdown enhanced pexophagy flux, as indicated by reduced p62 levels and increased LC3‐II/I ratio, whereas ATG5 co‐knockdown markedly reversed these changes (Figure 8d,e). Immunofluorescence staining further demonstrated that PMP70 were decreased in USP30 knockdown cells but re‐accumulated in the double knockdown cells, with maximal accumulation observed in ATG5‐deficient cells (Figure 8f). Consistently, quantitative analysis confirmed that the reduction in peroxisome abundance and morphological abnormalities observed after USP30 knockdown was restored upon ATG5 depletion (Figure 8g,h). Collectively, these findings indicate that ATG5 is indispensable for USP30 knockdown induced peroxisomal clearance, supporting that pexophagy is required for maintaining peroxisomal homeostasis under HG conditions.

## 3 Discussion

Peroxisomal dysfunction in proximal tubular cells plays a crucial role in the pathogenesis of DKD. It is essential to clarify the molecular mechanisms underlying tubular cell injury in order to identify new therapeutic targets for DKD. In this study, we identify USP30 as a key deubiquitinating enzyme for PEX5 and characterizes their expression and ubiquitination status both in vivo and in vitro. It demonstrates that PEX5 ubiquitination is an essential step for pexophagy, promoting the degradation of damaged peroxisomes during DKD progression. Mechanistically, the reduction of USP30 expression results in the degradation of ubiquitinated PEX5 on peroxisomes and induction of catalase that upregulates autophagosome formation. These two cellular consequences of USP30 loss cooperatively promote the degradation of peroxisomes by autophagy. Moreover, deletion of the essential autophagy factor ATG5 abolished the protective effects of USP30 knockdown. Together, our findings support USP30 as a novel regulator of pexophagy in DKD.

Recently, evidence suggests that peroxisomes are not only passive organelles but may contribute to metabolic disturbance and oxidative stress during diabetic kidney injury. Wang et al. showed that, in STZ‐induced diabetic mice, peroxisome‐related metabolism promotes succinate accumulation, which is associated with renal lipid deposition and increased ROS [18]. However, the underlying relationship between peroxisome abundance and downstream pexophagy is not fully defined. Our study showed that in human DKD kidney tissues, db/db mouse kidneys, and HG‐induced HK‐2 cells, increased levels of peroxisomal markers (PMP70), together with abnormal morphology and distribution. This change is more likely to reflect abnormal accumulation with altered structure, or a compensatory increase in peroxisome number accompanied by reduced quality, rather than the enhancement of peroxisomal function [19]. One study showed that, in SARS‐CoV‐2‐infected cells, peroxisomal membrane undergo the disruption and rearrangement of its structure, leading to the aberrant peroxisome morphology [20]. Moreover, under cellular stress, increased peroxisome abundance may initially represent a compensatory response to mitigate lipid overload and ROS. However, when pexophagy is impaired, dysfunctional peroxisomes may accumulate and further aggravate oxidative stress and lipotoxicity [21, 22].

The proper import of peroxisomal matrix proteins is fundamental to peroxisomal function. A number of peroxins (PEX) genes participate in the process of peroxisome biogenesis of these proteins [23, 24]. For this reason, we further examined key components of the matrix protein import machinery—including PEX3, PEX5, PEX14, and PEX19, which are closely tied to peroxisome quality control and the initiation of pexophagy. Our results indicate that reduced pexophagy in DKD may be associated with the altered regulation of PEX5 and PEX14 protein levels. However, PEX3 and PEX19 did not show significant differences; this possibly because PEX13 is rapid loss, making changes in its abundance difficult to detect [25]. Importantly, it is well established that Pex5 monoubiquitination at K209 triggers pexophagy under oxidative stress [26]. Omics profiling and immunoprecipitation analysis further revealed that PEX5 ubiquitination is reduced in DKD, suggesting that dysregulated activity of ubiquitination or deubiquitination enzymes may be involved in modulating PEX5 recycle and matrix protein import.

In recent years, growing evidence showed that deubiquitination enzyme (DUBs) play a critical role in the progression of DKD. For example, OTUD5 alleviates podocyte injury by reduces K63‐linked polyubiquitination of TAK1 at residue K158 and inhibiting the TAK1‐mediated inflammatory response in DKD [27]. USP22 could affect the process of DKD by deubiquitinating and stabilizing Sirt1 in glomerular mesangial cells [28].Similarly, a novel, specific inhibitor of USP30 (MXT652) could be used to protected against tubular atrophy and fibrosis in models of ischemia‐reperfusion injury and unilateral ureter obstruction [29]. Considering this, we performed Co‐IP analysis in HG‐induced HK‐2 cells and confirmed the interact between USP30 and PEX5. The colocalization of USP30 with the peroxisomal marker PMP70 supported the presence of a peroxisome‐associated USP30 expression in renal tubular epithelial cells, consistent with previous evidence that USP30 can localize independently to peroxisomes and regulate basal pexophagy [15]. USP30 knockdown in diabetic mice alleviated tubular injury and DKD progression whereas overexpression of USP30 in RTECs aggravated RTECs injury in both diabetic mice and HK‐2 cells. The possible mechanism for this progress could be high‐glucose‐induced oxidative stress promote USP30 dysregulation, thereby enhancing PEX5 deubiquitination and suppressing the clearance of damaged peroxisomes. The accumulation of dysfunctional peroxisomes further increase ROS production and tubular cell injury [30].

Notably, our study characterizes the expression pattern of USP30 in the kidney, demonstrating its predominant localization in renal tubular cells and a minimal expression in the glomeruli. Our findings strengthen its critical pathological significance in the kidney and highlight USP30 inhibition as a promising therapeutic strategy for DKD. Nevertheless, since USP30 also regulates mitochondrial quality control and apoptosis, systemic USP30 inhibition may produce biological effects beyond pexophagy [31]. Future studies should also systematically evaluate the dose, duration, therapeutic window, and target selectivity of systemic USP30 inhibition.

During pexophagy, the targeted peroxisomes are isolated within autophagosomes and eventually fuse with lysosomes and being degraded [22]. Previous studies reveal a critical role of p62 and NBR1 in regulation of pexophagy under basal conditions [32]. The function of p62 and NBR1 in HG‐induced pexophagy requires further investigation. Treatment with downregulated NBR1 in PEX5‐KD cells, supporting the presence of NBR1 is required for pexophagy. Since pexophagy is a form of selective autophagy, we investigated whether inhibition of ATG5 could clarify the contribution of the autophagy machinery to pexophagy impairment in the context of DKD. We found that the degradation of peroxisomes by USP30 knockdown was completely blocked in ATG5 deficient HK‐2 cells. In addition, the reduction in peroxisomal protein levels and the improvement in tubular cell injury observed upon USP30 knockdown were both blocked by ATG5 knockdown. Consistent with our study, Jo et al. reported that loss of HSPA9 induces pexophagy via an ATG5‐dependent canonical autophagy pathway [33]. Although further studies are needed to elucidate the molecular mechanism of pexophagy in response to HG stimulation, the USP30‐PEX5 axis and ATG5 may contribute to peroxisome degradation in USP30‐KD cells.

Interestingly, we observed that PEX5 and PEX14 showed divergent regulation under DKD conditions, with increased PEX5 but persistent downregulation of PEX14 despite increased peroxisomal abundance. This pattern suggests that peroxisome accumulation under diabetic stress may not reflect fully functional peroxisome biogenesis, but rather an imbalance between peroxisomal membrane expansion and matrix protein import capacity. Because PEX14 is an essential component of the peroxisomal import machinery and is involved in catalase import into peroxisomes [34], its reduction may contribute to impaired peroxisomal antioxidant capacity. In addition, PEX14 has been implicated in OPTN‐mediated pexophagy [35], suggesting that altered PEX14 expression may also affect peroxisome quality control. In this study, we focused on the USP30‐PEX5 ubiquitination axis; however, the divergent regulation of PEX14 indicates that PEX14‐dependent import and pexophagy pathways may also participate in DKD‐associated peroxisomal dysfunction and need further investigation.

Although our study demonstrated the effects of USP30 on peroxisomal dysfunction in vivo and in vitro, there are still some limitations. First, the specific domains mediating the interaction between PEX5 and USP30, the precise underlying mechanism under DKD conditions, and the subcellular localization of USP30 in renal tubular epithelial cells require further investigation. Second, a specific USP30 inhibitor mice is needed to confirm these effects. Third, the sample size of human specimens was limited, and larger cohorts are needed to validate the association of USP30/PEX5 and peroxisomal abnormalities with disease stage and renal function parameters in DKD patients. Finally, renal tubule‐specific USP30 knockout models are needed to determine the tubular cell‐autonomous role of USP30 and further validate its contribution to tubular injury in DKD.

In conclusion, our findings identify USP30 as an important regulator of peroxisomal homeostasis in DKD. USP30 knockdown increased overall PEX5 ubiquitination and peroxisomal turnover, restored catalase expression, and alleviated tubular injury, whereas ATG5 knockdown attenuated these protective effects. These findings support a model in which USP30 upregulation impairs PEX5 ubiquitination‐dependent pexophagy, thereby promoting the accumulation of dysfunctional peroxisomes and tubular injury. Therefore, targeting USP30 may represent a promising strategy for restoring peroxisomal quality control in DKD.

## 4 Experimental Section

### 4.1 Human Specimens

This study protocol was approved by the Ethics Committee of the First Affiliated Hospital of Zhengzhou University (approval No. 2025‐KY‐2059) and was conducted in accordance with the principles of the Declaration of Helsinki. Written informed consent was obtained from all participants. Renal biopsy specimens were collected from patients with diabetic kidney disease (DKD) in the Department of Nephrology, the First Affiliated Hospital of Zhengzhou University.

Control kidney tissues were obtained from histologically normal, non‐tumorous regions of nephrectomy specimens. Individuals with a documented history of diabetes were excluded. The tissues were fixed in 4% formalin immediately after collection to minimize potential effects associated with ischemic time. The pathological staging of DKD was performed by two independent renal pathologists in a blinded manner, according to the Renal Pathology Society (RPS) classification system [36]. The clinical baseline characteristics of patients with DKD are listed in Table S1.

### 4.2 Animal Studies

All mouse experiments were conducted in accordance with institutional guidelines for the care and use of laboratory animals and were reported in accordance with the ARRIVE 2.0 guidelines. The animal study was approved by the Ethics Committee of the Experimental Animal Center of Zhengzhou University (approval No. Z‐2025‐09005). The db/db mice and their littermate db/m controls, USP30‐KO mice (C57BL/6NGpt‐Usp30^em127Cd64/Gpt, strain T001876) carrying a 64‐bp deletion in Usp30 generated using CRISPR/Cas9‐mediated genome editing were purchased directly as homozygous animals from GemPharmatech (Nanjing, China). Genotypes were confirmed by PCR followed by agarose gel electrophoresis, and loss of USP30 protein was verified by Western blotting. Blood glucose and body weight were measured every 2 weeks. Blood and urine samples were collected for subsequent analyses.

6‐week‐old male db/m mice were randomly divided into three groups: (1) control group (n = 6), fed a standard diet; (2) db/m + Vehicle group (n = 6), injected with the control AAV9 vector; and (3) db/m + USP30 overexpression (USP30‐OE) group (n = 6), injected with AAV9 carrying full‐length mouse Usp30. At 10 weeks of age, mice received a 100 µL tail‐vein injection of either AAV2/9‐Ksp‐cadherin‐m‐Usp30 (AAV9‐USP30; 1.9 × 10^1^ ^2^ vg/mL; total dose, 1.9 × 10^1^ ^1^vg per mouse) or AAV2/9‐Ksp‐cadherin‐m‐control vector (1.8 × 10^1^ ^2^ vg/mL; total dose, 1.8 × 10^1^ ^1^ vg per mouse). Tail‐vein injections were performed under inhalational anaesthesia with additional analgesia. The Ksp‐cadherin promoter was used to preferentially drive transgene expression in renal tubular epithelial cells. Renal tubular transduction were verified by immunofluorescence co‐staining of USP30 with E‐cadherin and by quantitative PCR analysis of USP30 expression in kidney tissue. AAV vectors were purchased from Hanbio (Shanghai, China).

6‐week‐old male WT and Usp30‐KO mice were randomly assigned to each groups (n = 6). After 3 weeks of high‐fat diet (HFD) feeding, mice were fasted for 12 h with free access to water. At 9 weeks of age, intraperitoneal injections of streptozotocin (STZ; 50 mg/kg/day) for five consecutive days. Random blood glucose was measured 7 days after the final STZ injection. Mice with random blood glucose levels >16.7 mmol/L were considered diabetic. The DKD model was confirmed by a significant increase in uACR relative to age‐matched non‐diabetic controls, together with renal histopathological change. All mice were continuously maintained on the HFD and euthanized at 4 and 12 weeks after STZ injection, and kidney tissues were harvested for further analyses.

### 4.3 Cell Culture and Treatment

Human proximal tubular epithelial cells (HK‐2) cells (ATCC, Manassas, VA, USA) were cultured in DMEM/F12 (Gibco; #11966‐025/#11765‐054) supplemented with 10% FBS (Gibco; #10099141) and 1% penicillin–streptomycin (Procell; #PB180120) at 37°C in a humidified incubator. Cells were treated with normal glucose (NG, 5.6 mM glucose) or high glucose (HG, 30 mM glucose) as indicated. USP30 overexpression or knockdown (Table S2) was achieved using lentiviral vectors (Hanbio, Shanghai, China). USP30 overexpression or knockdown was achieved using lentiviral vectors carrying a puromycin‐resistance cassette (Hanbio, Shanghai, China). HK‐2 cells were transduced at an MOI of 20 in the presence of 5 µg/mL polybrene, followed by selection with 5 µg/mL puromycin for 2 days. Transduction efficiency was further confirmed by qRT‐PCR and Western blotting (Figures S4 and S5). SiRNAs targeting ATG5, PEX3, PEX19, PEX5, PEX14, and NBR1, as well as the negative control siRNA, were synthesized by RiboBio (Guangzhou, China) (Table S3). Transfections were performed with Lipofectamine 3000 (Thermo Fisher Scientific; L3000008) according to the manufacturer's protocol. Bafilomycin A1 (MCE, HY‐100558) were used in the HG‐induced HK‐2 cells for 12 h. Cycloheximide (CST, #2112) were added in HG‐induced HK‐2 cells.

### 4.4 RNA Sequencing

Total RNA was extracted from renal cortex tissues using TRIzol reagent. Library preparation and sequencing were performed by Kangce biotechnological (Wuhan, China). Raw sequencing reads were quality‐filtered and trimmed using fastp. Clean reads were then aligned to the human reference genome using STAR (v2.7.6a). Gene and transcript level quantification was performed with featureCounts (Subread v1.5.1; Bioconductor). Gene expression levels were normalized and reported as FPKM. Differentially expressed genes (DEGs) between groups were identified using the R package edgeR, with significance thresholds of log_2_ *fold change* > 1 or < 0.5 and *p* < 0.05. GO and KEGG enrichment analyses of DEGs were conducted using KOBAS (v2.1.1), with a *p‐value* cutoff of 0.05 to define statistically significant enrichment.

### 4.5 Western Blotting

Kidney tissues or HK‐2 cells were lysed in RIPA buffer supplemented with 1% phenylmethylsulfonyl fluoride (PMSF), a protease inhibitor cocktail (CW2200S; CWBio, Beijing, China), and a phosphatase inhibitor cocktail (CW2383S; CWBio, Beijing, China). Protein concentrations were determined using a BCA assay kit (Solarbio, China). Equal amounts of protein were separated by SDS‐PAGE and transferred onto 0.45 µm PVDF membranes or nitrocellulose (NC) membranes. Membranes were blocked with 5% milk at room temperature for 1 h and then incubated with primary antibodies overnight at 4°C. After washing with TBST, membranes were incubated with HRP‐conjugated secondary antibodies for 1 h at room temperature. Protein signals were visualized using an ECL detection reagent (Tanon, USA) and captured with a FUSION FX imaging system (VILBER, China). Primary antibodies used in this study are listed in Table S4.

### 4.6 Co‐Immunoprecipitation

Co‐immunoprecipitation (Co‐IP) was performed using the Pierce Protein A/G Magnetic Beads Kit (#P2179S; Beyotime, China). Cells were lysed using the lysis buffer supplied with the kit, supplemented with protease inhibitor cocktail and PMSF at a 1:100 dilution, and 80 µL of each lysate was retained as the input control. After pre‐clearing with normal IgG‐bound magnetic beads, lysates were divided into IP and species‐matched IgG‐control groups and incubated with the indicated antibodies, followed by Protein A+G magnetic beads at 4°C overnight. Immunocomplexes were washed, eluted in 1× SDS loading buffer, and analyzed by Western blotting. Detailed experimental conditions and antibody information are provided in the Supplementary Methods and Table S4.

### 4.7 Histological Analysis and Immunohistochemical Staining

Kidney tissues were fixed in 4% paraformaldehyde for 24 h and subsequently embedded in paraffin. Paraffin sections (4 µm) were deparaffinized and rehydrated, followed by staining with H&E, PAS, and Masson's trichrome. For IHC staining, kidney sections were subjected to antigen retrieval using citrate buffer (pH 6.0) and then blocked with 5% bovine serum albumin (BSA). Sections were incubated with primary antibodies (Table S4) overnight at 4°C, followed by incubation with an enhanced enzyme conjugated secondary antibody at 37°C for 20 min on the next day. PASM staining was performed by PAS staining followed by silver counterstaining. All images were acquired using a slide scanning microscope (Axio Scan 7; Zeiss, Germany).

### 4.8 Transmission Electron Microscopy

Renal cortex tissues were trimmed into approximately 1 mm^3^ blocks, and HK‐2 cells grown on coverslips were immediately fixed in pre‐cooled 4% glutaraldehyde, followed by post‐fixation with 1% osmium tetroxide. After washing with PBS, samples were dehydrated through a graded acetone series and embedded in Epon 812 epoxy resin. Ultra‐thin sections were prepared and examined using a transmission electron microscope (HT7800; Hitachi, Japan) operated at an accelerating voltage of 80 kV. Images were analyzed using ImageJ software (version 1.54 g). Six randomly selected, non‐overlapping fields obtained at the same magnification from three independent samples were analyzed per group. Peroxisomes were identified based on the presence of a single limiting membrane and a homogeneous, finely granular matrix and were manually counted. Peroxisome size was assessed by cross‐sectional area, whereas shape was evaluated using circularity and aspect ratio. The fractional volume was estimated as the total peroxisomal area divided by the analyzed cytoplasmic area and expressed as a percentage. All measurements were performed by two investigators blinded to the experimental groups.

### 4.9 Immunofluorescence Staining

HK‐2 cells and frozen kidney sections were fixed with 4% paraformaldehyde for 30 min and permeabilized with 0.1% Triton X‐100 at room temperature for 10 min. For immunofluorescence staining, samples were blocked with 3% bovine serum albumin (BSA) and incubated with primary antibodies (Table S4) overnight at 4°C. Next day, samples were incubated with Alexa Fluor–conjugated secondary antibodies (Invitrogen, USA), counterstained with 4,6‐diamidino‐2‐phenylindole (DAPI). All images were acquired using a Zeiss LSM880 confocal microscope (Carl Zeiss, Germany).

### 4.10 Catalase Activity Assay

Catalase activity in HK‐2 cells was measured using a Catalase Assay Kit (#S0051; Beyotime, China) according to the manufacturer's instructions and normalized to the total protein concentration determined by the BCA assay. Results are expressed as U/mg protein.

### 4.11 Quantitative Real‐Time PCR

Total RNA was extracted from cultured cells and tissue samples using the RNeasy Mini Kit (Qiagen, Hilden, Germany) according to the manufacturer's instructions. cDNA was synthesized by reverse transcription using a commercial kit (RR036A; Takara, Japan). qRT‐PCR was performed using a SYBR Green PCR kit (RR820A; Takara, Japan) to determine the relative expression levels of target genes. All mRNA primers were designed and synthesized by Sangon Biotech (Shanghai, China) (Table S5).

### 4.12 Statistical Analysis

Statistical analyses were performed using GraphPad Prism 10 (GraphPad Software, Inc., La Jolla, CA, USA). Data are presented as the mean ± standard deviation (SD). Normality and homogeneity of variance were assessed using the Shapiro‐Wilk test and Levene's test, respectively. Differences between two groups were analyzed using an unpaired, two‐tailed Student's *t*‐test. For comparisons among multiple groups with a single variable, one‐way analysis of variance (ANOVA) followed by Tukey's post hoc test was applied. Two‐way ANOVA with Bonferroni's post hoc test was used to assess differences among groups involving two variables. For data that did not conform to a normal distribution, the Kruskal‐Wallis test was performed. The Correlation analyses were conducted by Spearman's correlation test. *p* value < 0.05 was considered statistically significant.

## Author Contributions

**Dr. Zhangsuo Liu**: and **Dr. Jiayu Duan**: designed the study; Dr. Jia Li conducted experiments, and wrote the manuscript. **Dr. Chaoyang Hua**: and **Dr. Furong Zhai**: collected the samples from murine models; **Dr. Shaokang Pan**: performed cell‐based experiments. **Dr. Jia Li**: and **Dr. Guangpu Liu**: drew the figures. **Dr. Dongwei Liu**: conducted statistical analysis and revised the manuscript. **Dr. Jianguo Wen**, **Dr. Zhangsuo Liu**: and **Dr. Jiayu Duan**: edited various versions of the manuscript. All authors approved the final version of the manuscript.

## Funding

This work was supported by grants from the Program of the Natural Science Foundation of Henan Province (No. 252300423858 and No. 252300423190).

## Ethics Statement

The human study was reviewed and approved by the Ethics Committee of the First Affiliated Hospital of Zhengzhou University (approval No. 2025‐KY‐2059). Written informed consent was obtained from all participants or their legal representatives, as appropriate. The animal study was approved by the Ethics Committee of the Experimental Animal Center of Zhengzhou University (approval No. Z‐2025‐09005).

## Conflicts of Interest

The authors have no conflict of interest to declare.

## Supporting information




**Supporting File 1**: advs77161‐sup‐0001‐SuppMat.docx.

**Supporting File 2**: advs77161‐sup‐0002‐SuppMat.docx.

**Supporting File 3**: advs77161‐sup‐0003‐SuppMat.xlsx.

**Supporting File 4**: advs77161‐sup‐0004‐SuppMat.docx.

## Data Availability

The data of this study are available from the corresponding author upon reasonable request.
