# Supplementary material for: USP30‐Mediated Deubiquitination of PEX5 Suppresses Pexophagy to Drive Tubular Injury in Diabetic Kidney Disease
Source: Adv Sci (Weinh). 2026 Aug 21:e77161. Online ahead of print. doi: 10.1002/advs.77161 (PMC13496153; doi:10.1002/advs.77161)
Supplement: Supplementary file 2 [file ADVS-9999-e77161-s002.docx]

**Supplementary Figures**

**Figure S1**

**Figure S1** Peroxisome accumulation is increased in RTECs of patients with DKD and db/db mice. **(a)** Schematic diagram of the sampling collection procedures of the mouse model. **(b)** kidney weight-to-body weight ratio. n=6 mice/group. (**c)** Representative images of H&E staining in kidney tissues from db/db and db/m mice. **(d)** Representative H&E, Masson, PASM and PAS staining in renal biopsies from patients with DKD. Black scale bar, 20µm. **(e)** Representative immunofluorescent staining for Synaptopodin (green) and PMP70 (red) in the kidney tissue from db/db and db/m mice (DAPI, blue). Scale bar, 10µm. (**f, g)** quantification of PMP70 and E-cadherin expression in db/db and db/m mice from Figure 1l. n=6. **(h)** RNA sequencing analysis of the expression of PMP70 gene in kidney tissues from db/db and db/m mice. n=6. **(i)** The expression of PMP70 mRNA levels in db/db and db/m mice was analyzed by qRT-PCR. n=6. Data are presented as means ± SD. Statistical significance was determined by two-tailed unpaired Student’s *t*-test. **P*<0.05, ***P*< 0.01, ****P*<0.001, *****P*<0.0001, ns, not significant.

**Figure S2**

**FigureS2** Accumulated peroxisomes in RTECs exhibit functional impairment and defective pexophagy. **(a)** Quantification of PPARγ protein levels in HK-2 cells treated with HG for different durations. **(b, c)** The expression of PEX3 and PEX19 mRNA levels was analyzed by qRT-PCR in HK-2 cells. n=3. **(d)** Western blot analysis of PEX3 levels in HK-2 cells. n=3. **(e, f)** Quantitative analysis of peroxisome density and representative immunofluorescent staining for PMP70 (red) in siPEX3 and siCTRL groups. Scale bar, 10µm. n=3. **(g)** Western blot analysis of PEX19 levels in HK-2 cells. n=3. **(h, i)** Quantitative analysis of peroxisome density and representative immunofluorescent staining for PMP70 (red) in siPEX19 and siCTRL groups. Scale bar, 10µm. n=3. **(j, k)** Catalase activity in siPEX5 and siPEX14 groups. Catalase activity was normalized to total protein concentration and expressed as U/mg protein. **(l)** The expression of NBR1 mRNA levels was analyzed by qRT-PCR in HK-2 cells. n=3. **(m)** Representative immunofluorescent staining for PMP70 (red) and NBR1 (green) in the siPEX5 and siCTRL groups. Scale bar, 10µm. **(n)** Representative immunofluorescent staining for LAMP1 (red) and PMP70 (green) in the NG and HG groups. Scale bar, 10µm. **(o, p)** Western blot and quantification of the autophagy-related protein levels in HK-2 cells treated with HG for different durations. Data are shown as means ± SD and were analyzed by one-way ANOVA followed by Tukey’s post-hoc test for multiple pairwise comparisons. **P*<0.05, ***P*< 0.01, ****P*<0.001, *****P*<0.0001 ns, not significant.

**Figure S3**

**Figure S3** Upregulation of USP30 is associated with reduced ubiquitination-dependent PEX5 degradation. **(a)** KEGG analysis of the biological processes involved in the predicted transcriptional pathways. **(b)** Schematic diagram of the treatment and sampling collection procedures of the mouse model. **(c-f)** Body weight, uACR, blood glucose and kidney weight in db/db and db/m mice. n=6 mice/group. **(g-l)** Quantification of PMP70, USP30, PEX5, Catalase, KIM-1 and E-cadherin levels in db/db and db/m mice. n=6. Data are presented as means ± SD. Statistical analyses for Figure S3g to l were performed using two-way ANOVA followed by Bonferroni’s post-hoc test for pairwise comparisons. **P*< 0.05, ***P*< 0.01, ****P*<0.001, *****P*<0.0001.

**Figure S4**

**Figure S4** Overexpression of USP30 exacerbates peroxisome accumulation and impairs pexophagy in db/m mice. **(a)** The diagram for vector atlas of USP30 overexpression AAV harboring Ksp-cadherin promoter. **(b)** kidney weight in db/m mice. n=6 mice/group. **(c)** The expression of USP30 mRNA levels was analyzed by qRT-PCR. n=6. **(d)** Representative images of Masson staining and PAS staining in kidney tissues from db/m mice. n=6. Red scale bar, 20µm. **(e-j)** Quantification of PMP70, USP30, PEX5, Catalase, KIM-1 and E-cadherin levels in db/m mice. n=6. **(k-n)** Quantification of Atg5, NBR1, p62, and LC3 II/I ratio levels in db/m mice. n=6. **(o)** Quantification of Catalase relative intensity in the immunofluorescent images shown in (p). **(p)** Representative immunofluorescent staining for Catalase (red) and E-cadherin (green) in the kidney tissues from db/m mice (DAPI, blue). Scale bar, 10µm. Data are shown as means ± SD and were analyzed by two-way ANOVA followed by Bonferroni’s post-hoc test for pairwise comparisons. ***P*< 0.01, ****P*<0.001, *****P*<0.0001.

**Figure S5**

**Figure S5** USP30 knockout alleviates tubular injury in DKD mice by restoring peroxisomal function and facilitating pexophagy. **(a)** Representative agarose gel electrophoresis images to verify genotyping in USP30-KO mice. **(b)** Representative images of Masson staining and PAS staining in kidney tissues from DKD mice. Red scale bar, 20µm. **(c)** kidney weight in DKD mice. n=6 mice/group. **(d)** The expression of USP30 mRNA levels was analyzed by qRT-PCR. n=6. **(e-j)** Quantification of PMP70, USP30, PEX5, Catalase, KIM-1 and E-cadherin levels in DKD mice. n=6. **(k-n)** Quantification of Atg5, NBR1, p62, and LC3 II/I ratio levels in DKD mice. n=6. **(o)** Quantification of Catalase relative intensity in the immunofluorescent images shown in (p). **(p)** Representative immunofluorescent staining for Catalase (red) and E-cadherin (green) in the kidney tissues from DKD mice (DAPI, blue). Scale bar, 10µm. Data are shown as means ± SD and were analyzed by two-way ANOVA followed by Bonferroni’s post-hoc test for pairwise comparisons. **P*< 0.05, ***P*< 0.01, ****P*<0.001, *****P*<0.0001.

**Figure S6**

**Figure S6** USP30 knockdown enhances pexophagy and ameliorates tubular injury in HG-induced HK-2 cells. **(a, b)** The expression of USP30 and PMP70 mRNA levels was analyzed by qRT-PCR. n=3 independent biological replicates. **(c)** Western blot analysis of USP30, PMP70, KIM-1, and Catalase expression in HK-2 cells treated with HG under the indicated conditions. **(d)** Quantification of Atg5, p62, and LC3 II/I ratio levels in HK-2 cells under the indicated conditions. n=3 independent biological replicates. **(e, f)** Quantification of mean peroxisome number and fractional volume in the Figure 6j. Six images from different microscopic fields were analyzed in each group. Data are shown as means ± SD and were analyzed by one-way ANOVA followed by Tukey’s post-hoc test in Figure S6a, S6b and S6d. Statistical analyses for Figure S6e and S6f were performed using two-way ANOVA followed by Bonferroni’s post-hoc test for pairwise comparisons. **P*< 0.05, ***P*< 0.01, ****P*<0.001, *****P*<0.0001, ns, not significant.

**Figure S7**

**Figure S7** USP30 overexpression impaired pexophagy and aggravated tubular injury in HK-2 cells. (a) The expression of USP30 mRNA levels was analyzed by qRT-PCR. n=3. (b, c) Quantification of mean peroxisome number and fractional volume in the Figure 7a. Six images from different microscopic fields were analyzed in each group. Data are presented as means ± SD. Statistical analyses were performed using two-way ANOVA followed by Bonferroni’s post-hoc test for pairwise comparisons. **P*< 0.05, ***P*< 0.01, ****P*<0.001, *****P*<0.0001, ns, not significant.
