# Supplementary material for: USP30‐Mediated Deubiquitination of PEX5 Suppresses Pexophagy to Drive Tubular Injury in Diabetic Kidney Disease
Source: Adv Sci (Weinh). 2026 Aug 21:e77161. Online ahead of print. doi: 10.1002/advs.77161 (PMC13496153; doi:10.1002/advs.77161)
Supplement: Supplementary file 3 [file ADVS-9999-e77161-s003.docx]

**Supplementary Methods**

- **Animal Studies**

Mice were housed under specific pathogen-free conditions with a 12-h light/dark cycle at [22 ± 2°C] and [50–60%] humidity, with free access to food and water. Animal experiments were reported in accordance with the ARRIVE 2.0 guidelines. AAV tail-vein injections were performed under inhalational anesthesia. STZ was administered intraperitoneally by trained personnel without anesthesia. Because both procedures involved brief, nonsurgical needle injections, no additional analgesics were administered, in accordance with the approved animal protocol. Animals were monitored daily for signs of pain or distress. Humane endpoints included body-weight loss exceeding 20%, severe dehydration, persistent recumbency, impaired access to food or water, labored breathing, or other signs of severe distress. Animals meeting these criteria were euthanized immediately.

- **Co-immunoprecipitation**

Co-immunoprecipitation (Co-IP) was performed using the Pierce Protein A/G Magnetic Beads Kit (#P2179S; Beyotime, China). Cells were lysed using the lysis buffer supplied with the kit, supplemented with protease inhibitor cocktail and PMSF at a 1:100 dilution. Approximately 80μL of each lysate was retained as the input control, and 1 mL of lysate was used for immunoprecipitation.

For pre-clearing, 10μL of Protein A+G magnetic beads were washed twice with 1× TBS and incubated with 1μg normal IgG for 1h at room temperature with gentle rotation. The IgG-bound beads were washed three times with 1×TBS and then incubated with the cell lysate for 1h at 4°C to remove nonspecifically bound proteins. After magnetic separation, the pre-cleared lysate was divided equally into the IP and IgG-control groups.

Each 500μL aliquot was incubated with the indicated IP antibody or normal species-matched IgG for 2-3h at 4 °C with gentle rotation. IP antibodies were used at 1-2μg per 100-500μg of total protein, according to the antibody-specific conditions listed in Supplementary Table S4. Subsequently, approximately 15μL of washed Protein A+G magnetic beads was added, and the samples were incubated overnight at 4 °C with gentle rotation.

The immunocomplexes were collected using a magnetic separator and washed three times. Bound proteins were eluted in 30-50μL of 1×SDS loading buffer by heating at 99°C for 10min and subsequently analyzed by Western blotting. For immunoblotting, membranes were incubated with primary antibodies overnight at 4°C with gentle agitation, followed by the corresponding secondary antibodies for 1h at room temperature. Antibody sources, catalogue numbers, host species, and application-specific working dilutions or amounts are provided in Supplementary Table S4.

- **Sequences of lentivirus (USP30 overexpression)**

atgctgagctcccgggccgaggcggcgatgaccgcggccgacagggccatccagcgcttcctgcggaccggggcggccgtcagatataaagtcatgaagaactggggagttataggtggaattgctgctgctcttgcagcaggaatatatgttatttggggtcccattacagaaagaaagaagcgtagaaaagggcttgtgcctggccttgttaatttagggaacacctgcttcatgaactccctgctacaaggcctgtctgcctgtcctgctttcatcaggtggctggaagagttcacctcccagtactccagggatcagaaggagcccccctcacaccagtatttatccttaacactcttgcaccttctgaaagccttgtcctgccaagaagttactgatgatgaggtcttagatgcaagctgcttgttggatgtcttaagaatgtacagatggcagatctcatcatttgaagaacaggatgctcacgaattattccatgtcattacctcgtcattggaagatgagcgagaccgccagcctcgggtcacacatttgtttgatgtgcattccctggagcagcagtcagaaataactcccaaacaaattacctgccgcacaagagggtcacctcaccctacatccaatcactggaagtctcaacatccttttcatggaagactcactagtaatatggtctgcaaacactgtgaacaccagagtcctgttcgatttgatacctttgatagcctttcactaagtattccagccgccacatggggtcacccattgaccctggaccactgccttcaccacttcatctcatcagaatcagtgcgggatgttgtgtgtgacaactgtacaaagattgaagccaagggaacgttgaacggggaaaaggtggaacaccagaggaccacttttgttaaacagttaaaactagggaagctccctcagtgtctctgcatccacctacagcggctgagctggtccagccacggcacgcctctgaagcggcatgagcacgtgcagttcaatgagttcctgatgatggacatttacaagtaccacctccttggacataaacctagtcaacacaaccctaaactgaacaagaacccagggcctacactggagctgcaggatgggccgggagcccccacaccagttctgaatcagccaggggcccccaaaacacagatttttatgaatggcgcctgctccccatctttattgccaacgctgtcagcgccgatgcccttccctctcccagttgttcccgactacagctcctccacatacctcttccggctgatggcagttgtcgtccaccatggagacatgcactctggacactttgtcacttaccgacggtccccaccttctgccaggaaccctctctcaactagcaatcagtggctgtgggtctccgatgacactgtccgcaaggccagcctgcaggaggtcctgtcctccagcgcctacctgctgttctacgagcgcgtcctttccaggatgcagcaccagagccaggagtgcaagtctgaagaaTACCCCTATGACGTGCCAGACTACGCCTATCCTTACGATGTCCCCGATTATGCTTACCCATACGACGTTCCAGACTACGCAtga
