# Supplementary material for: USP30‐Mediated Deubiquitination of PEX5 Suppresses Pexophagy to Drive Tubular Injury in Diabetic Kidney Disease
Source: Adv Sci (Weinh). 2026 Aug 21:e77161. Online ahead of print. doi: 10.1002/advs.77161 (PMC13496153; doi:10.1002/advs.77161)
Supplement: Supplementary file 4 [file ADVS-9999-e77161-s001.docx]

**Figure 1**

**Figure 2**

**Figure S2**

**Figure 3**

**Figure 4**

**Figure 5**

**Figure 6**

**Figure S6**

**Figure 7**

**Figure 8**
